# The selective orexin receptor 1 antagonist ACT-335827 in a rat model of diet-induced obesity associated with metabolic syndrome

**DOI:** 10.3389/fphar.2013.00165

**Published:** 2013-12-30

**Authors:** Michel A. Steiner, Carla Sciarretta, Anne Pasquali, Francois Jenck

**Affiliations:** ^1^CNS Pharmacology Neurobiology, Actelion Pharmaceuticals Ltd.Allschwil, Switzerland; ^2^Immunology, Actelion Pharmaceuticals Ltd.Allschwil, Switzerland; ^3^Cardiology, Actelion Pharmaceuticals Ltd.Allschwil, Switzerland

**Keywords:** diet-induced obesity, metabolic syndrome, food intake, orexin, orexin receptor antagonist, ACT-335827, food preference, lipid metabolism

## Abstract

The orexin system regulates feeding, nutrient metabolism and energy homeostasis. Acute pharmacological blockade of orexin receptor 1 (OXR-1) in rodents induces satiety and reduces normal and palatable food intake. Genetic OXR-1 deletion in mice improves hyperglycemia under high-fat (HF) diet conditions. Here we investigated the effects of chronic treatment with the novel selective OXR-1 antagonist ACT-335827 in a rat model of diet-induced obesity (DIO) associated with metabolic syndrome (MetS). Rats were fed either standard chow (SC) or a cafeteria (CAF) diet comprised of intermittent human snacks and a constant free choice between a HF/sweet (HF/S) diet and SC for 13 weeks. Thereafter the SC group was treated with vehicle (for 4 weeks) and the CAF group was divided into a vehicle and an ACT-335827 treatment group. Energy and water intake, food preference, and indicators of MetS (abdominal obesity, glucose homeostasis, plasma lipids, and blood pressure) were monitored. Hippocampus-dependent memory, which can be impaired by DIO, was assessed. CAF diet fed rats treated with ACT-335827 consumed less of the HF/S diet and more of the SC, but did not change their snack or total kcal intake compared to vehicle-treated rats. ACT-335827 increased water intake and the high-density lipoprotein associated cholesterol proportion of total circulating cholesterol. ACT-335827 slightly increased body weight gain (4% vs. controls) and feed efficiency in the absence of hyperphagia. These effects were not associated with significant changes in the elevated fasting glucose and triglyceride (TG) plasma levels, glucose intolerance, elevated blood pressure, and adiposity due to CAF diet consumption. Neither CAF diet consumption alone nor ACT-335827 affected memory. In conclusion, the main metabolic characteristics associated with DIO and MetS in rats remained unaffected by chronic ACT-335827 treatment, suggesting that pharmacological OXR-1 blockade has minimal impact in this model.

## Introduction

Metabolic syndrome (MetS) represents a group of risk factors for diabetes and cardiovascular disease; it is characterized by abdominal obesity plus two of four factors including: elevated circulating triglyceride (TG) levels, reduced circulating high density lipoprotein-associated cholesterol (HDLc) levels, elevated blood pressure, and elevated fasting plasma glucose levels (International Diabetes Federation; http://www.idf.org). Diet-induced obesity (DIO), which is a major causative factor of MetS, can also result in cognitive impairments and in poorer hippocampus-dependent memory function (Eskelinen et al., 2008; Francis and Stevenson, 2011). The current primary intervention for MetS is to lower body weight through reduced energy consumption, a change of diet composition, and increased physical activity (De Flines and Scheen, 2010). Pharmacological treatments are expected to additionally help patients to restrict over-eating by reducing systemic and brain signals responsible for driving high caloric palatable food intake (Adamo and Tesson, 2008).

Orexin neuropeptides A and B modulate energy balance and metabolic homeostasis (Sakurai, 2006) but also palatable food and sweet reward perception (Di Sebastiano and Coolen, 2012). Orexins are expressed by a discrete number of neurons in the lateral hypothalamus (De Lecea et al., 1998; Sakurai et al., 1998) and their effects are mediated by the G-protein-coupled orexin receptors type 1 and 2. OXRs are expressed widely in orexinergic projection areas throughout the brain including the mesolimbic system, implicated in reward/aversion, and including several mid- and hindbrain regions, implicated in regulating energy homeostasis and food intake (Trivedi et al., 1998; Marcus et al., 2001). For instance, some orexin neurons project to the ventral tegmental area (VTA), where they synapse with dopaminergic and GABAergic neurons (Balcita-Pedicino and Sesack, 2007); other orexin neurons innervate neuropeptide Y and pro-opiomelanocortin expressing neurons of the arcuate nucleus (Niimi et al., 2001; Tsuneki et al., 2010). Orexin neurons also receive multiple afferent input (Yoshida et al., 2006) from cortico-limbic regions, involved in either positive or negative emotional processing, as well as from local hypothalamic areas, involved in defense reactions, in autonomic and in circadian regulation (Furudono et al., 2006); in addition, orexin neuron activation is modulated by the anorectic adipokine leptin, by the orectic peptide ghrelin, by circulating non-essential amino acids, and by falling glucose levels (Yamanaka et al., 2003; Williams et al., 2008; Louis et al., 2010; Karnani and Burdakov, 2011). OXR mRNA or protein is also found in the periphery including enteric neurons, stomach, pancreas, adrenal gland, and white and brown adipose tissue (Kirchgessner and Liu, 1999; Blanco et al., 2002; Digby et al., 2006; Adeghate et al., 2010; Skrzypski et al., 2011). The relative functional significance of orexins in controlling energy homeostasis and food intake by acting both in the brain and periphery (Heinonen et al., 2008) has remained elusive but orexin peptide or OXR levels appear to be regulated under a variety of nutritional conditions such as food deprivation, sweetener-induced overconsumption and genetic obesity (Mondal et al., 1999; Karteris et al., 2005; Furudono et al., 2006).

The preferential OXR-1 antagonist SB-334867 has been described to reduce normal food intake in rats during the dark phase (Haynes et al., 2000) by decreasing meal size (Parise et al., 2011) and enhancing satiety onset (Ishii et al., 2005). In addition to impacting quantitative natural feeding the OXR-1 may play a key role in mediating the hedonic component of nutrient intake. SB-334867 centrally injected in the VTA of rats inhibited feeding stimulated by intra-nucleus accumbens injections of a mu-opioid receptor agonist (Zheng et al., 2007). The systemic administration of SB-334867 reduced the overconsumption of a HF diet in satiated rats (Choi et al., 2010), and another OXR-1 antagonist, GSK1059865, reduced the binge-like intake of a HF/S diet (Piccoli et al., 2012). Chronic administration of a high dose of SB-334867 to ob/ob female mice lacking leptin reduced food intake and weight gain (Haynes et al., 2002), and this effect was associated with improvements in glucose homeostasis. Contrarily, OXR-1 deficient mice became similarly obese to wild-type mice when challenged with a HF diet (Funato et al., 2009). However, consistent with the reported effects of chronic SB-334867 treatment, fasting blood glucose and serum insulin levels were improved in obese OXR-1 deficient mice (Funato et al., 2009). Finally, a series of studies exploring SB-334867 in operant responding models also suggest that blocking OXR-1 signaling can reduce the motivation to consume HF/S foods (Borgland et al., 2009; Choi et al., 2010; Cason and Aston-Jones, 2013). Central infusion of the OXR-2 specific agonist [Ala11,D-Leu15]Orexin-B over a period of 14 days decreased consumption of a HF diet and reduced DIO (Funato et al., 2009). Genetically elevating brain levels of orexins also prevented DIO by decreasing food intake and stimulating energy expenditure through a mechanism requiring OXR-2. Therefore, either blocking OXR-1 or activating OXR-2 signaling may provide benefits under conditions of prolonged access to high palatable food that will lead to DIO.

The purpose of our study was to test the effects of the novel OXR-1 antagonist ACT-335827 (Steiner et al., 2013) in a rat model of DIO associated with MetS. In comparison to the frequently used OXR-1 antagonist SB-334867, which may also bind to other neuronal receptors (Gotter et al., 2012; Morairty et al., 2012), ACT-335827 shows greater selectivity among a panel of more than 100 neuronal targets [it only binds to the melatonin MT_1_ receptor with greater than 50% (i.e., 58%) inhibition at 10μM; (Steiner et al., 2013)]. Affinity of ACT-335827, measured by an intracellular calcium release assay, at the recombinant rat OXR-1 was between 7 and 25 nM (depending on incubation time) and between 630 and 1030 nM at the OXR-2. The calculated free brain concentration in Wistar rats after oral gavage of 300 mg/kg was between 97 nM at 1 h and 166 nM at 6 h following administration. Accordingly, the oral dose of 300 mg/kg was effective in reducing a number of anxiety and stress-related measures in the rat including autonomic activation, schedule-induced polydipsia, and fear-potentiated startle; similar to dual OXR antagonists (Steiner et al., 2012). However, in contrast to dual OXR antagonists, ACT-335827 did not simultaneously induce sleep or hypolocomotion at 300 mg/kg, likely because it did not block OXR-2 at his dose (Steiner et al., 2013).

In the present study MetS was induced in rats exposed to a CAF diet with constant access and free choice between HF/S diet or SC. In addition, highly caloric human snacks were provided 4 times weekly. Once metabolic disturbances were established (13 weeks), rats were treated daily before the onset of the dark phase with 300 mg/kg of ACT-335827 or vehicle for 4 weeks. The effects of chronic ACT-335827 treatment were monitored on energy and water intake, food preference, abdominal obesity, glucose homeostasis, plasma lipid levels, and blood pressure. Hippocampus-dependent cognitive function, which has been shown to be impaired in rodents exposed to DIO (Hwang et al., 2010; Davidson et al., 2012), was also assessed in parallel using a contextual fear-conditioning paradigm.

## Materials and methods

### Animals

Thirty male Wistar rats (Harlan, Horst, NL) weighing 160–180 g (6–7 weeks old) were single-housed upon arrival and acclimated to the Actelion animal facility under a regular 12 h light/dark cycle (lights on at 6:00) for 2 weeks. During this time they were fed SC [Diet 2018S: 18% kcal fat, 24% kcal protein, 58% kcal carbohydrate (CHO) and 3.1 kcal/g; Harlan Teklad Diets, Madison, WI, USA] *ad libitum*. All experimental procedures were performed in strict accordance with the relevant licenses issued by the Basel-Land Veterinary office and adhered to Swiss federal regulations on animal experimentation.

### Diets

After acclimatization, rats were randomly divided into two groups. One group of 10 rats (250–325 g; 8–9 weeks old) continued to receive SC *ad libitum* and served as the control group for the effects of CAF diet feeding. A second group of 20 rats (275–320 g) was given a CAF diet comprised of a free choice between SC and a HF/S diet (Diet 2126: 47% kcal fat, 19.5% kcal protein, 33.5% kcal CHO and 4.6 kcal/g; Provimi Kliba, Kaiseraugst, Switzerland), both provided *ad libitum*. The CAF diet group also received a human snack food 4 days per week of 15 kcal (weeks 1–5 of diet exposure), 30 kcal (weeks 6–13 of diet exposure), or *ad libitum* for 1 h at the beginning of the dark phase (weeks 14 to 18 of diet exposure). Seven types of snacks were provided (see Supplementary Table 1). One snack type was given per day and they were alternated between sweet and savory. The inclusion of human snacks, which are both highly caloric and high in sugar, fat, and salt, can be an important addition to rodent models of DIO, since voluntary over-snacking by humans may potentiate obesity, as well as glycemic and cardiovascular abnormalities (Kimokoti and Brown, 2011; Sampey et al., 2011).

### Experimental design

**Figure 1:**
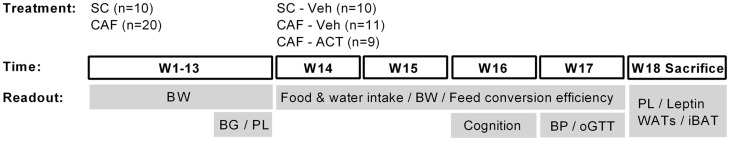
**Schematic overview of the experimental setup.** Rats were maintained on standard chow (SC; *n* = 10) or cafeteria diet (CAF; *n* = 20) for 13 weeks (W). The CAF diet was comprised of a free choice between SC and a high fat/sweet diet, both provided at libitum, and intermittent additional access to human snacks. During these 13 weeks body weight (BW) was measured weekly and blood glucose (BG) and plasma lipid (PL) concentrations were assessed in week 11 and 13, respectively. Chronic vehicle (Veh) or ACT-335827 treatment (ACT; 300 mg/kg, p.o., once daily before onset of the dark phase) started in week 14. All SC fed rats received vehicle treatment only (SC-Veh; *n* = 10). The CAF diet fed rats were divided into a vehicle (CAF-Veh; *n* = 11) and an ACT-335827 (CAF-ACT; *n* = 9) treatment group based on an even distribution of their blood glucose and plasma triglyceride levels, measured in week 11 and 13. During weeks 14–17 the food and water intake and the body weight gain was assessed, and the feed conversion efficiency calculated. In week 16 cognitive hippocampal function was assessed using a contextual fear paradigm. In week 17 blood pressure (BP) measurements and an oral glucose tolerance test (oGTT) were performed. In the beginning of week 18 all rats were sacrificed. Their plasma lipid (PL) and plasma leptin levels were assessed and the weight of white adipose tissue deposits (WATs) and the interscapular brown adipose tissue (iBAT) deposit was measured. See the Methods for further details.

Please see Figure 1 for a schematic outline of the experimental design.

Both groups of animals (SC or CAF fed) were maintained on their respective diets for 13 weeks and their body weights were monitored once weekly. Blood glucose and plasma lipids levels were determined at the end of week 11, after a 16 h fast, and at the beginning of week 13, after a 6–8 h fast, respectively.

Chronic ACT-335827 or vehicle treatment began in week 14 of diet exposure. The CAF-diet fed rats were evenly assigned to a vehicle (CAF-Veh, *n* = 11) or an ACT-335827 (CAF-ACT, *n* = 9) treatment group based on their fasting blood glucose levels obtained in week 11. All SC fed rats received vehicle treatment only (SC-Veh, *n* = 10). ACT-335827 or vehicle was orally administered daily, 2 h before the onset of the dark phase. Body weights were recorded in the afternoon before the start of treatment and once weekly thereafter. Food and water intake in the home cages were continuously measured by an automated system (Phenomaster, TSE systems, Bad Hamburg, Germany).

In week 3 of treatment (week 16 of diet exposure), rats were tested in a contextual fear conditioning (CFC) paradigm. At the beginning of week 4 of treatment (week 17 of diet exposure), blood pressure of the rats was measured in the morning. At the end of the same week, the rats were food deprived (16 h) before an oral glucose tolerance test (oGTT) in the morning. At the beginning of week 18 of diet exposure, the rats were sacrificed by decapitation 24 h after the last ACT-335827 administration and following a mild food deprivation (4–6 h).

### Drugs

ACT-335827 hydrobromide (Actelion Pharmaceuticals Ltd., Switzerland) was freshly prepared in 10% polyethylene glycol 400/0.5% methylcellulose in water, which served as vehicle (Veh). It was administered orally at 300 mg/kg based on the weight of the free base, in a volume of 5 mL/kg, and administered daily 2 h before the onset of the dark phase.

The 300 mg/kg dose was chosen based on the pharmacokinetics of this compound, which were outlined in the Introduction. At this dose, calculated free brain concentrations (>97nM) were sufficient to allow a significant blockade of OXR-1 (affinity at the rat receptor: 7–25 nM, depending on the assay), without yet blocking the OXR-2 (affinity: 630–1030 nM). This was confirmed by the pharmacological actions of this compound, i.e., reducing stress-related outcomes without inducing sleep at this dose. Following oral administration of ACT-335827 at 300 mg/kg, calculated free brain concentrations were rising from 1 h (97 nM) to 6 h (166 nM), and declined thereafter (1 nM at 24 h). Rats consumed the majority of their food during the active, dark phase, and they increased their eating pattern just before the switch of the light-dark cycle. Thus, the rats were treated 2 h before onset of the dark phase, and also the snacks (the most caloric type of food used in this experiment) were provided at the beginning of the dark phase, in order to assure that the large majority of food intake during this chronic study would take place under full blockade of the OXR-1.

### Fasting blood glucose and plasma lipid analysis before chronic drug treatment

In week 11 of diet exposure, rats were fasted overnight (16 h). Using gentle restraint, a tail blood sample was obtained and glucose levels were determined using a Glucometer (ACCU-CHEK, Roche Diagnostics, Germany). For plasma lipid analysis in week 13, rats were mildly fasted (6–8 h) and blood samples were obtained by sublingual vein puncture under isoflurane anesthesia, divided between EDTA or heparin containing microtainer tubes, and kept on ice. Plasma was collected by centrifugation (2400 × g for 10 min at 4°C), placed in pre-chilled tubes and stored at −20°C. Levels of non-esterified fatty acid (NEFA), total cholesterol, and TG were assayed in plasma from EDTA-mixed blood, whereas HDL-cholesterol (HDLc) was quantified in plasma from heparinized blood. All lipids were measured using commercial enzyme assay kits and a fully automated analyzer (Beckman Coulter AU480, Nyon, Switzerland).

### Contextual fear conditioning (CFC)

A single-training trial CFC paradigm was used to assess the effects of the CAF diet in combination with ACT-335827 treatment on hippocampus-dependent learning addressing simple contextual memory. For the training of conditioned fear, rats were singly placed in lit transparent plastic boxes (27 × 27 × 40 cm) equipped with stainless steel grid shock floors within an enclosed cubicle (Ugo Basile, Comerio, Italy) for 5 min. A 1 mA footshook was then delivered for 2 s. After 2 min the rats were returned to their home cages. 24 h later, rats were re-exposed to the same box for a 10 min testing phase. Freezing behavior (defined as absence of all movements except breathing) was measured using an automated video-tracking system (Anymaze; Stoelting Co., Wood Dale, IL, USA).

### Blood pressure

Blood pressure was indirectly measured by blood volume pressure recording (VPR) from the tail using the CODA 8-channel high throughput non-invasive blood pressure system (EMKA Technologies, Paris, France). Animals were pre-warmed at 37°C. They were then placed in restraining tubes and body temperature was maintained using a heating pad. An occlusion tail-cuff was inserted at the base of the tail and a transducer was placed 1 cm below. Rats underwent 7 cycles of measurements, lasting ~5 min, before they were returned to their home cage. Following manual reviewing, at least 3 of the 7 measurements derived from clearly defined and smooth VPRs were selected for further analysis. Based on this criteria, two CAF diet fed rats treated with ACT-335827, and one CAF diet fed rat treated with vehicle, were excluded from analyses. The systolic and diastolic blood pressures were derived by taking the mean of the 3 selected values. The mean blood pressure was calculated using the following formula: (systolic pressure - diastolic pressure)/3 + diastolic pressure.

### Oral glucose tolerance test (oGTT)

An oGTT was performed between 8:00 and 12:00 at the end of the fourth week of treatment (week 17 of diet exposure) on overnight fasted rats (16 h). In the morning, tail vein blood was collected from rats of all treatment groups in random order for determination of fasting glucose levels (as described above). Approximately 30 min later, a freshly prepared solution of glucose (2 g/kg/5 mL in water) was orally administered to the rats in a staggered manner, and blood was again collected for glucose quantification 30, 60, 120, and 180 min following oral glucose administration.

### Tissue and blood collection at sacrifice

Eighteen to Twenty hours after the last ACT-335827 or vehicle administration, rats were mildly fasted for 4–6 h before sacrifice by decapitation. Trunk blood was collected, divided between EDTA- and heparin- containing tubes, and kept on ice for ~2 h before centrifugation. Plasma was collected into pre-chilled tubes and stored at −20°C for lipid quantification (as described above) or at −80°C for leptin analysis by a commercially available leptin detection assay (Mesoscale, Gaithersburg, USA). Epididymal, abdominopelvic, and mesenteric white adipose tissues (WATs), as well as intrascapular brown adipose tissue (iBAT) were then rapidly isolated and weighed.

### Statistical analysis

For each rat, food intake in grams was converted to energy intake in kcal. For the time course analysis of energy and water intake and diet preference, kcal values or water volumes (mL) were cumulated over either 48 h or weekly. Body weight gain was expressed as the percentage change from baseline weight at the start of diet exposure or from the beginning of chronic treatment. The area under the curve (AUC) of the time course of blood glucose changes for each rat during the oGTT was calculated using the trapezoid rule. The weekly feed efficiency of each rat was determined by normalizing body weight gain to the total kcal intake of a given week.

For all measures, mean values and standard errors of the mean (s.e.m.) were calculated per treatment/diet group. Before the beginning of chronic treatment, the blood glucose and plasma TG levels of the CAF diet groups were compared to each other and to those of the SC fed group using unpaired two-tailed *t*-tests, except for diet preference and weight gain, which was analyzed using a Two-Way analysis of variance (ANOVA). Following the start of chronic vehicle or ACT-335827 treatment, for all readouts the effects of CAF diet feeding *per se* were determined by comparing CAF diet fed vehicle treated rats with SC fed vehicle treated rats using unpaired *t*-tests or Two-Way ANOVAs. The effects of ACT-335827 were also determined for all readouts by comparing vehicle-treated and ACT-335827-treated CAF fed rats using unpaired *t*-tests, and Two-Way or Three-Way ANOVAs where appropriate. CAF fed ACT-335827-treated rats were not compared to SC-fed vehicle-treated rats. *Post-hoc* mean comparisons were made using Tukey or Bonferroni tests. All statistical analyses were performed using GraphPad Prism 5.03 or Statistica (version 9) software, and statistical significance was accepted at *p* < 0.05.

## Results

### Profile of diet groups before treatment onset

**Figure 2:**
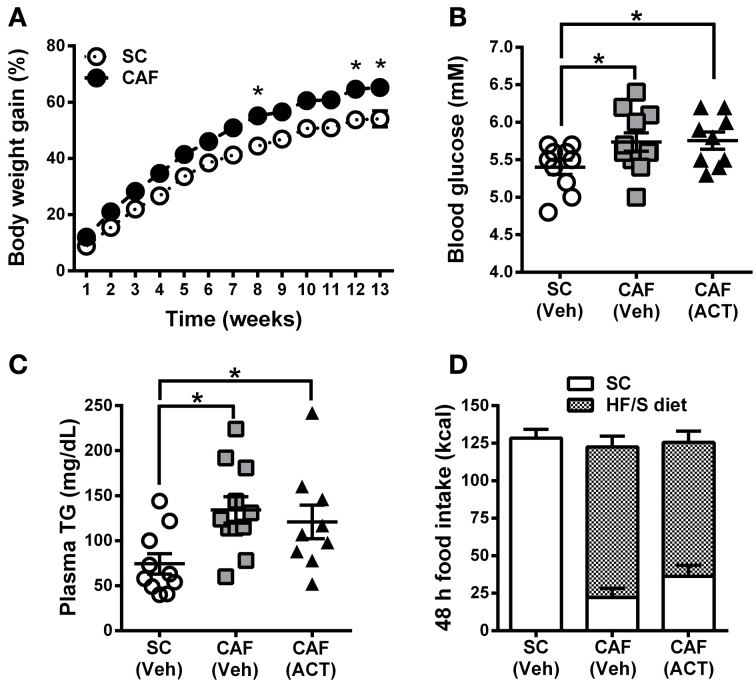
**Weight gain, MetS and food intake before treatment onset. (A)** Time course of body weight gain over 13 weeks of SC and CAF diet feeding. Body weight gain is expressed as the percentage change from baseline body weight at the start of diet exposure. Mean ± s.e.m. (SC *n* = 10; CAF *n* = 20). ^*^p < 0.05 vs. SC at the indicated time point by *post-hoc* test following ANOVA. Levels of whole blood glucose **(B)**, and of plasma triglycerides (TG) **(C)** were analyzed in week 11 and 13, respectively. The group of 20 CAF diet fed rats was subdivided into one Veh (*n* = 11) and one ACT (*n* = 9) group which were going to start receiving treatment (Veh and ACT indicated in brackets) in week 14. Horizontal bars represent the mean ± s.e.m. ^*^p < 0.05 by *t*-test. **(D)** Energy intake and food preference cumulated over 48 h before the start of Veh or ACT treatment. No snack was given during this 48 h period. All three groups consumed a similar amount of kcal. Both of the CAF diet fed groups preferred the HF/S diet over the SC and they did not differ statistically in their level of preference. Mean + s.e.m. (*n* = 9–11) per group. (*SC*, standard chow; *CAF*, cafeteria diet; *HF/S*, high fat/sweet; *Veh*, vehicle; *ACT*, ACT-335827).

Over the 13 weeks of diet exposure, the body weight gain of CAF diet fed rats was significantly greater than that of SC fed rats [*Diet*: *F*_(1, 28)_ = 12.20, *p* < 0.05; *Time × Diet*: *F*_(12, 336)_ = 3.42, *p* < 0.05; Figure 2A]. *Post-hoc* analyses revealed significant group differences in weeks 8, 12, and 13 of diet exposure (Figure 2A).

Fasting glucose levels of CAF diet fed rats were significantly elevated at week 11 when compared to SC fed rats [*t*_(30)_ = 2.3, *p* < 0.05]. This parameter was used to divide CAF diet fed rats in two subgroups with a similar mean and distribution of glucose levels (Figure 2B) that were then assigned to receive either vehicle or ACT-335827 upon the start of treatment in week 14. Both subgroups showed similar significantly elevated fasting glucose levels compared to SC fed rats [CAF(Veh) vs. SC: *t*_(19)_ = 2.14, *p* < 0.05; CAF(ACT) vs. SC: *t*_(17)_ = 2.39, *p* < 0.05; CAF(Veh) vs. CAF(ACT): *t*_(18)_ = 0.11, *p* = 0.91].

At week 13 of diet exposure plasma TG levels were also found elevated in both groups fed a CAF diet as compared to SC [CAF(Veh) vs. SC: *t*_(19)_ = 3.18, *p* < 0.01; CAF(ACT) vs. SC: *t*_(17)_ = 2.18, *p* < 0.05] (Figure 2C). TG levels did not differ between the CAF diet fed groups [*t*_(18)_ = 0.57, *p* = 0.56]. Plasma levels of NEFA, total cholesterol, HDLc, or the proportion of HDLc in total cholesterol were not elevated in CAF diet fed rats compared to SC diet fed rats (data not shown).

Finally, before the start of chronic drug treatment in week 14, a 48 h assessment of the total energy intake of the HF/S diet and SC on days when no snacks were presented (Figure 2D) was performed. The two groups of rats exposed to the CAF diet and the rats fed SC did not differ in their total kcal intake. With respect to food choice, both of the CAF diet fed groups consumed significantly more of the HF/S diet (77.3% of total calories) than SC (22.7% of total calories) [*Food*: *F*_(1, 18)_ = 53.11, *p* < 0.05], but they did not differ in their level of preference [*CAF diet* treatment *Group × Food*: *F*_(1, 18)_ = 1.97, *p* = 0.18]. With respect to water intake, the three groups of rats did not significantly differ (Supplementary Figure 1A).

In summary, rats fed the CAF diet gained more weight and showed elevated fasting blood glucose and plasma TG levels compared to rats fed SC. Forty-eight hours before the onset of chronic ACT-335827 or vehicle administration, the two CAF diet fed groups did not differ from each other in their total kcal and water intake and in their preference of the HF/S diet over SC.

### ACT-335827 reduces the preference for a HF/S diet without affecting weekly total energy intake, and increases body weight gain and feed conversion efficiency

**Figure 3:**
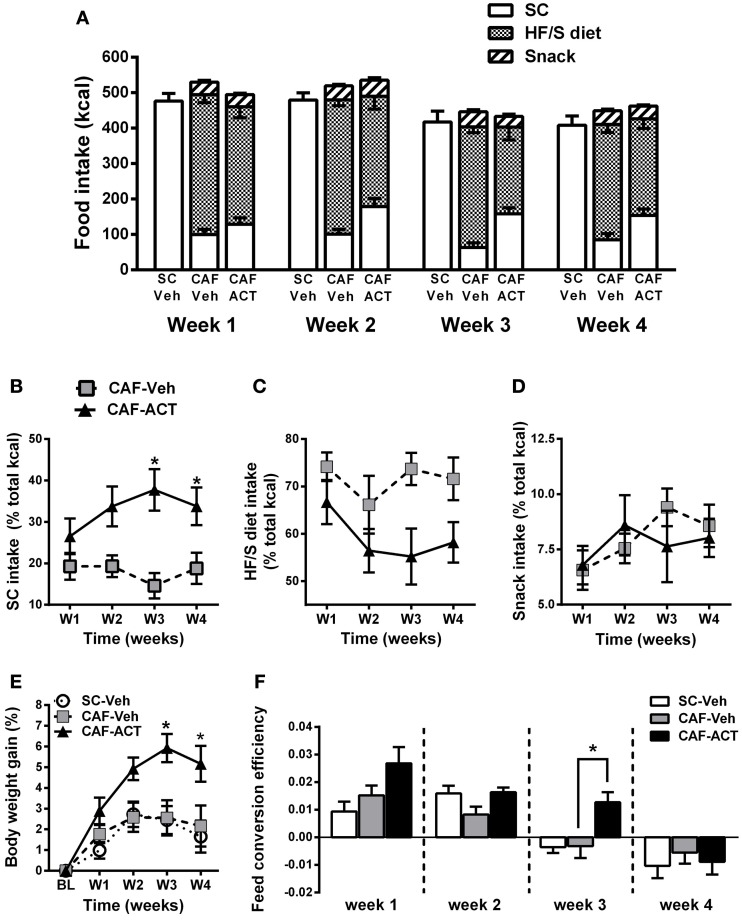
**Effect of diet and chronic ACT-335827 treatment on feeding behavior, body weight, and feed efficiency. (A)** Total energy intake divided into SC, HF/S diet, and snack intake cumulated weekly. CAF-diet fed Rats chronically treated with ACT-335827 reduced their preference for the HF/S diet over SC. Snack intake was not affected by ACT-335827. For further statistical analyses see Results. **(B–D)** Energy intake of SC, HF/S diet, and snack expressed as the percentage of the total energy intake and cumulated in 1 week time bins over the 4 weeks (weeks 14–17) of Veh or ACT treatment. Significant differences between groups were revealed only for the SC intake, not for the HF/S diet or snack intake. ^*^p < 0.05 vs. CAF-Veh at the indicated time points by *post-hoc* test following ANOVA. **(E)** Body weight gain expressed as a percentage change from baseline (BL) weight (i.e., at start of chronic treatment). CAF-Veh and SC-Veh groups did not statistically differ from each other. ^*^p < 0.05 vs. CAF-Veh at the indicated time points by *post-hoc* test following ANOVA. **(F)** Weekly efficiency of conversion of total kcal intake into body weight gain. No significant differences were revealed between the SC-Veh and CAF-Veh groups. Mean ± s.e.m. (*n* = 9–11) per group. ^*^p < 0.05 vs. CAF-Veh at the indicated time points by *post-hoc* test following ANOVA. Note that throughout this manuscript SC-Veh is always compared to CAF-Veh, and CAF-ACT is always compared to CAF-Veh. No comparisons between CAF-ACT and SC-Veh were made. (*SC*, standard chow; *CAF*, cafeteria diet; *Veh*, vehicle; *ACT*, ACT-335827; *HF*/*S*, high fat/sweet).

In general, all three experimental groups slightly reduced their total energy intake over the 4 weeks of vehicle or ACT-335827 treatment [SC-Veh vs. CAF-Veh: *Time*: *F*_(3, 57)_ = 17.2, *p* < 0.05; CAF-Veh vs. CAF-ACT: *Time*: *F*_(3, 34)_ = 21.57, *p* < 0.05; Figure 3A]. Vehicle treated rats fed a CAF diet did not differ in their weekly total kcal intake compared to vehicle treated rats fed SC [*Diet*: *F*_(1, 19)_ = 3.13, *p* = 0.09; *Diet × Time*: *F*_(3, 57)_ = 0.26, *p* = 0.85]. Chronic administration of ACT-335827 did not affect the total kcal intake of the CAF diet compared to vehicle treatment [*Treatment*: *F*_(1, 18)_ = 0.001, *p* = 0.98; *Treatment × Time*: *F*_(3, 54)_ = 0.92, *p* = 0.44]. Both vehicle and ACT-335827 treated rats fed the CAF diet consumed significantly more of the HF/S diet than SC or snack [*Food*: *F*_(2, 36)_ = 157.4, *p* < 0.05]. Analysis of the contribution of each food type to the total kcal intake averaged over 4 weeks of vehicle treatment revealed that the rats obtained in average 74% of their total kcal from the HF/S diet, 18% from SC, and 8% from snacks. Rats chronically treated with ACT-335827 reduced their preference for the HF/S diet over SC [*Treatment × Food*: *F*_(2, 36)_ = 8.22, *p* < 0.05], irrespective of week of treatment [*Treatment × Food × Time*: *F*_(6, 108)_ = 0.13, *p* = 1.28].

More detailed analyses of the weekly energy intake of each food type separately over the 4 weeks of treatment revealed that ACT-335827 significantly increased the intake of SC as compared to vehicle [*Treatment*: *F*_(1, 18)_ = 11.24, *p* < 0.05], and this effect became stronger over time [*Treatment × Time*: *F*_(3, 54)_ = 3.08, *p* < 0.05; Figure 3B]. Indeed, *post-hoc* comparisons showed that ACT-335827 treatment significantly increased SC intake in weeks 3 and 4. Conversely, ACT-335827 treatment significantly decreased the intake of the HF/S diet as compared to vehicle [*Treatment*: *F*_(1, 18)_ = 7.47, *p* < 0.05], irrespective of week of treatment [*Treatment × Time*: *F*_(3, 54)_ = 0.742, *p* = 0.53; Figure 3C]. Finally, both the vehicle and ACT-335827 treated rats consumed a similar amount of the snack food over the 4 weeks of treatment [*Treatment*: *F*_(3, 54)_ = 1.38, *p* = 0.26; *Time*: *F*_(3, 54)_ = 2.66, *p* = 0.06; *Treatment × Time*: *F*_(3, 54)_ = 1.38, *p* = 0.25; Figure 3D].

Vehicle treated CAF diet fed rats and SC fed rats did not differ in their weight gain over the 4 weeks of treatment [*Time*: *F*_(4, 19)_ = 14.91, *p* < 0.05; *Diet × Time*: *F*_(1, 76)_ = 0.48, *p* = 0.74; Figure 3E]. In the CAF diet fed groups, chronic ACT-335827 treatment significantly increased weight gain over 4 weeks compared to vehicle treatment [*Treatment*: *F*_(1, 18)_ = 6.29, *p* < 0.05; *Treatment × Time*: *F*_(4, 72)_ = 5.42, *p* < 0.05]. *Post-hoc* analysis revealed that this increase was due to significantly greater weight gains by the ACT-335827 treated rats in weeks 3 and 4.

Normalization of weekly energy intake to absolute body weight revealed that CAF diet fed rats treated with vehicle were not hyperphagic compared to SC fed rats or to ACT-335827 treated rats over the 4 weeks of treatment (data not shown). However, examination of the efficiency of feed conversion into body weight gain of vehicle treated CAF diet fed and SC fed rats revealed a significant reduction for both groups over 4 weeks of treatment from a positive efficiency to a negative efficiency [*Time*: *F*_(3, 19)_ = 21.9, *p* < 0.05], but no effect of diet and no diet × time interaction [*Diet*: *F*_(1, 19)_ = 0.07, *p* = 0.80; *Diet × Time*: *F*_(3, 57)_ = 1.88, *p* = 0.14; Figure 3F]. Similarly, both groups of CAF fed rats showed decreasing feed efficiency over the 4 weeks of treatment [*Time*: *F*_(3, 19)_ = 22.4, *p* < 0.05]. However, ACT-335827 treatment inhibited this decrease compared to vehicle treatment [*Treatment*: *F*_(1, 19)_ = 5.00, *p* < 0.05], which was more pronounced during the first 3 weeks [*Treatment × Time*: *F*_(3, 54)_ = 2.65, *p* = 0.06] and reached significance in week 3 (*p* < 0.05; *post-hoc* test).

In summary, rats consuming a CAF diet and chronically treated with ACT-335827 slightly decreased their preference for a HF/S diet over SC without changing their weekly total kcal intake and snack consumption. CAF diet fed rats treated with ACT-335827 gained significantly more weight over 4 weeks compared to vehicle treatment, likely due to increased feed conversion efficiency.

### ACT-335827 increases water intake in CAF diet fed rats

Similar to the total food intake, overall water intake slightly decreased over the 4 weeks of treatment with vehicle or ACT-335827 independent of diet [SC-Veh vs. CAF-Veh: *Time*: *F*_(3, 57)_ = 18.91, *p* < 0.05; CAF-Veh vs. CAF-ACT: *Time*: *F*_(3, 54)_ = 11.3, *p* < 0.05; Supplementary Figure 1B]. Furthermore, vehicle treated rats fed CAF diet drank significantly less water than vehicle treated rats fed only SC, independent of week of treatment [*Diet*: *F*_(1, 19)_ = 10.95, *p* < 0.05; *Diet × Time*: *F*_(3, 57)_ = 0.03, *p* = 0.93]. In CAF fed rats, ACT-335827 treatment significantly increased water intake compared to vehicle treatment, largely irrespective of the week of administration [*Treatment*: *F*_(1, 18)_ = 12.39, *p* < 0.05; *Treatment × Time*: *F*_(3, 54)_ = 2.38, *p* = 0.08]. The effect reached statistical significance in the second and third week of treatment (*p* < 0.05; *post-hoc* test).

### No detectable emotional or cognitive impairments due to CAF diet exposure alone or in combination with chronic act-335827 treatment

Consumption of energy rich diets high in fat and/or sugar has been shown to impair learning and memory, particularly through their effects on hippocampal function. A CFC task was performed after 3 weeks of chronic ACT-335827 or vehicle treatment (17 weeks following the onset of CAF diet exposure).

**Figure 4:**
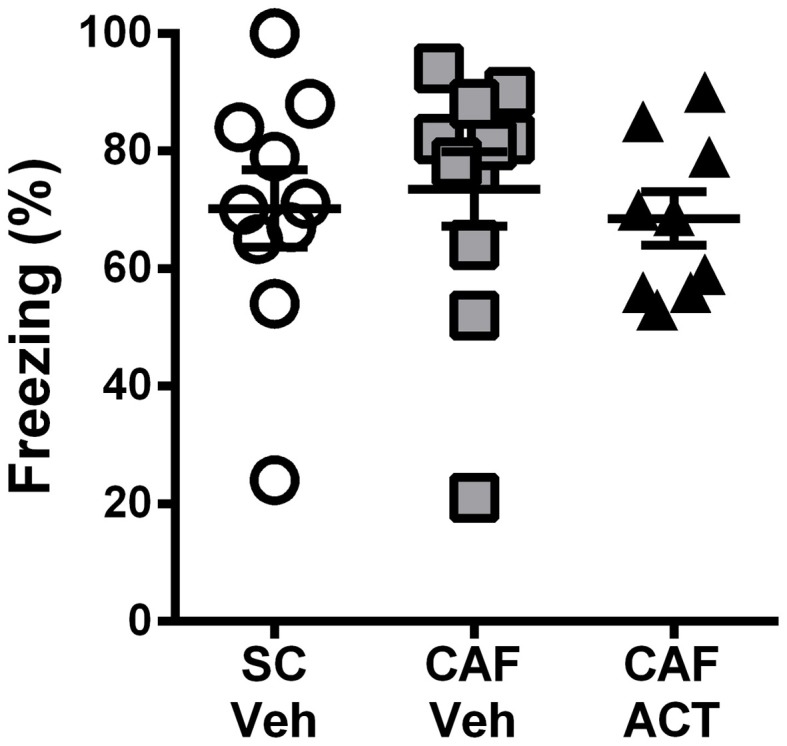
**Effect of CAF diet and ACT-335827 on cognition in week 16.** Percentage of time spent freezing over a 10 min test of contextual conditioned fear. Mean ± s.e.m. (*n* = 9–11 per group). No statistical difference between groups was revealed. (*SC*, standard chow; *CAF*, cafeteria diet; *Veh*, vehicle; *ACT*, ACT-335827).

During the fear conditioning training the rats of all groups responded with a similar amount of freezing over the 2 min following the presentation of a 1 mA foot shock (data not shown). When returned to the aversive conditioned context 24 h later, vehicle treated CAF-fed rats displayed a high level of freezing (~70%) over the entire 10 min of testing that was similar to that of both the vehicle treated rats fed SC [*t*_(19)_ = 0.37, *p* = 0.72] and the ACT335827 treated rats fed a CAF diet [*t*_(18)_ = 0.61, *p* = 0.55; Figure 4].

### CAF diet-induced glucose intolerance is not affected by act-335827 treatment

**Figure 5:**
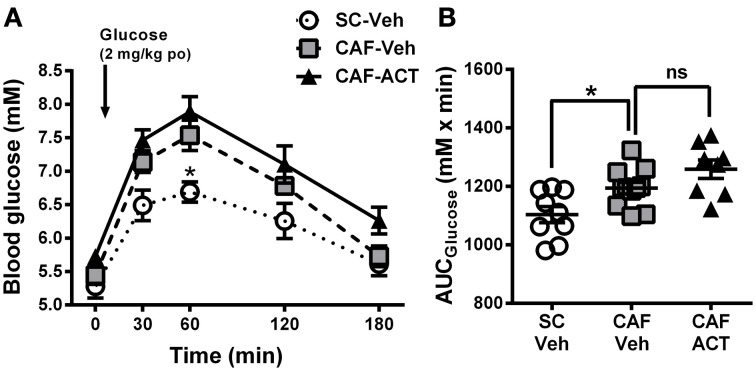
**Glucose homeostasis in week 17. (A)** Oral glucose tolerance test (oGTT) performed on overnight fasted SC and CAF fed rats treated with Veh or ACT. Whole blood glucose was measured before (0 min) and at the indicated time points after the oral (po) administration of 2 mg/kg glucose (arrow). ^*^p < 0.05 vs. CAF-Veh at the indicated time point by *post-hoc* test following ANOVA. Mean ± s.e.m. (*n* = 8–11) per group. Due to an incomplete blood sample set, data from one SC-Veh and one CAF-Veh rat had to be omitted because of repeated measures within subjects analyses. **(B)** AUC calculated individually for each rat. ^*^p < 0.05 by *t*-test. The CAF-ACT group was statistically not different from the CAF-Veh group. (*SC*, standard chow; *CAF*, cafeteria diet; *Veh*, vehicle; *ACT*, ACT-335827; *po*, per os; *AUC*, area under the curve; *ns*, not significant).

The effect of CAF diet exposure alone and in combination with ACT-335827 treatment on glucose homeostasis and tolerance were assessed. Following a 16 h fast, the blood glucose levels of vehicle-treated CAF fed rats were slightly elevated from those of SC fed vehicle treated rats, but the difference was not significant [*t*_(18)_ = 0.77, *p* = 0.45; Figure 5A]. In addition, ACT-335827 treatment of rats fed a CAF diet did not alter fasting glucose levels compared to vehicle treatment [*t*_(18)_ = 1.91, *p* = 0.07].

Upon oral glucose challenge, blood glucose levels were significantly elevated in vehicle treated CAF fed rats compared to SC fed rats [*Diet*: *F*_(2, 25)_ = 9.85, *p* < 0.05; Figure 5A], but the shape of the time course up to 180 min after challenge was not affected [*Diet × Time*: *F*_(8, 100)_ = 1.33, *p* = 0.24]. *Post-hoc* analyses revealed that the effect was most pronounced at the glucose peak (60 min after challenge), which was significantly higher in CAF fed rats than in SC fed rats. Analysis of the AUC_glucose_ confirmed the glucose intolerance of the CAF-fed rats compared to SC-fed rats [*t*_(18)_ = 2.72, *p* < 0.05]. Chronic ACT-335827 treatment in combination with CAF diet feeding showed a trend to impair glucose tolerance but did not significantly alter the levels or time course of blood glucose elevations upon challenge [*Treatment*: *F*_(1, 17)_ = 4.28, *p* = 0.054; *Treatment × Time*: *F*_(4, 68)_ = 0.23, *p* = 0.92; Figure 5B], or the AUC_glucose_ [*t*_(17)_ = 1.80, *p* = 0.09].

### CAF diet-induced elevation in blood pressure is not altered by chronic act-335827 treatment

**Figure 6:**
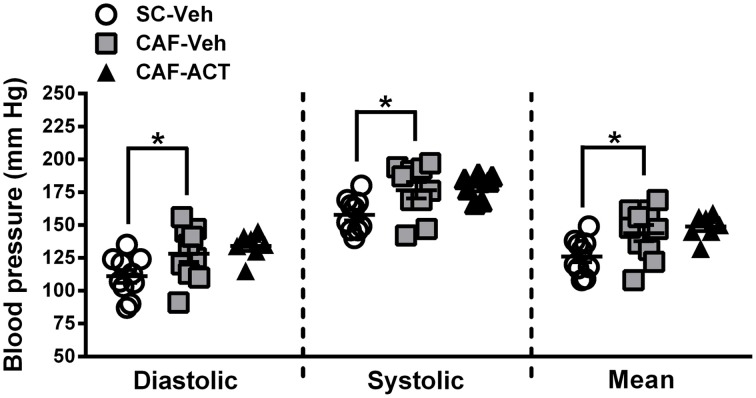
**Blood pressure in week 17.** Diastolic, systolic, and mean blood pressures of SC or CAF diet fed rats treated with Veh or ACT. Horizontal bars represent the mean ± s.e.m. (*n* = 7–10) per group. Reliable data was not attainable from 1 CAF-Veh rat and from 2 CAF-ACT rats, and were therefore omitted from analyses. ^*^p < 0.05 by *t*-test. No statistical difference was revealed between the CAF-ACT and the CAF-Veh group. (*SC*, standard chow; *CAF*, cafeteria diet; *Veh*, vehicle; *ACT*, ACT-335827).

Hypertension is correlated with central obesity and is an aspect of MetS. Compared to vehicle treated rats fed a SC diet, vehicle treated rats consuming a CAF diet for 17 weeks had significantly elevated diastolic [*t*_(18)_ = 2.16, *p* < 0.05], systolic [*t*_(18)_ = 2.53, *p* < 0.05], and mean blood pressure [*t*_(18)_ = 2.37, *p* < 0.05; Figure 6]. Chronic ACT-335827 treatment did not affect the elevation in blood pressure due to CAF diet feeding.

### ACT-335827 does not affect elevations in WAT and iBAT mass due to a CAF diet

**Figure 7:**
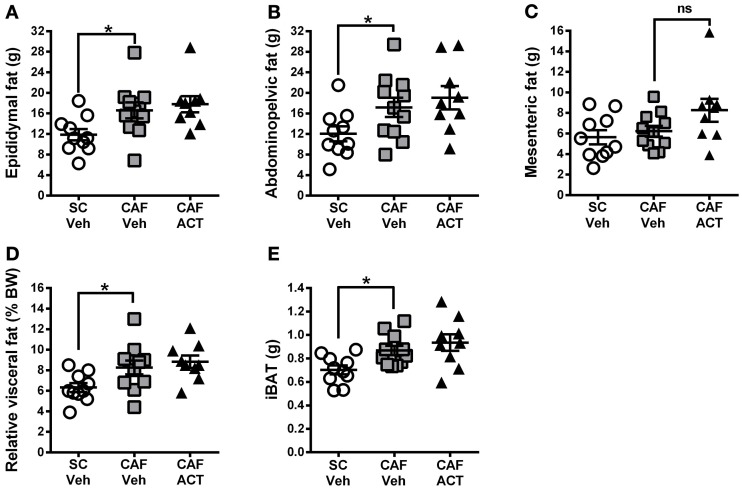
**Fat mass at sacrifice.** The effect of SC or CAF diet exposure on the mass of epididymal **(A)**, abdominopelvic **(B)**, and mesenteric **(C)** visceral fat deposits in rats treated with Veh or ACT. **(D)** Normalization of cumulated visceral fat mass (in **A–C**) to body weight (BW) per rat and expressed as a percentage. **(E)** Mass of intrascapular brown adipose tissue (iBAT). Mean ± s.e.m. (*n* = 9–11 per group). ^*^p < 0.05 by *t*-test. No statistical difference was revealed between the CAF-ACT and the CAF-Veh group. (*SC*, standard chow; *CAF*, cafeteria diet; *Veh*, vehicle; *ACT*, ACT-335827; *ns*, not significant).

Elevations in visceral white adipose tissue mass are associated with central obesity and are an indicator of MetS. The main visceral fat deposits in male rodents are the epididymal depot, the adominopelvic depot, and the mesenteric depot. At the time of sacrifice, following 4 weeks of chronic vehicle treatment, CAF diet fed rats had significantly heavier epididymal [*t*_(19)_ = 2.43, *p* < 0.05; Figure 7A] and adominopelvic [*t*_(19)_ = 2.13, *p* < 0.05; Figure 7B], but not mesenteric (Figure 7C) fat deposits, than SC fed rats. Moreover, the proportion of body weight represented by the combined mass of all three visceral fat deposits was significantly higher in vehicle treated rats fed a CAF diet compared to those fed SC [*t*_(19)_ = 2.36, *p* < 0.05; Figure 7D]. In addition to WAT, the CAF diet fed rats treated with vehicle also had significantly larger deposits of iBAT compared to vehicle treated SC fed rats [*t*_(19)_ = 3.0, *p* < 0.05; Figure 7E]. Chronic treatment with ACT-335827 did not significantly alter the elevations in visceral WAT depots and iBAT mass resulting from CAF diet feeding as compared to vehicle treatment.

### ACT-335827 increases the proportion of plasma HDLc levels, but does not alter elevated plasma TG levels due to a CAF diet

**Figure 8:**
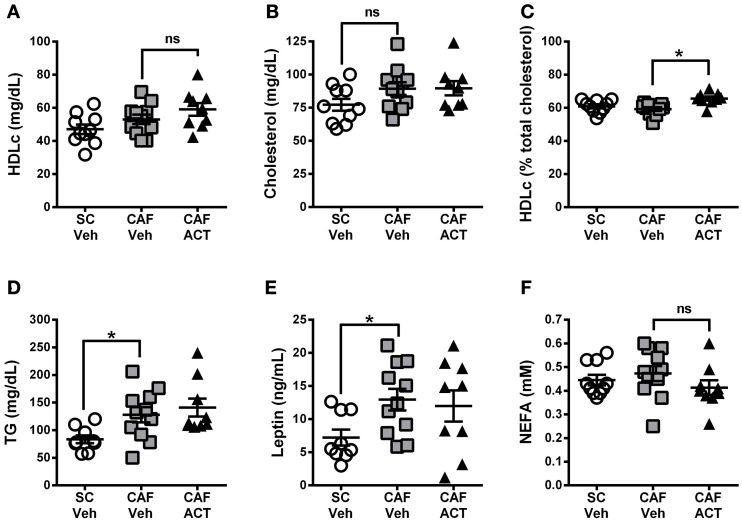
**Plasma lipids and leptin at sacrifice.** Plasma levels of HDL-cholesterol (HDLc) **(A)**, total cholesterol **(B)**, HDL-c levels (in **A**) expressed as a percentage of total plasma cholesterol (in **B**) **(C)**, triglyceride (TG) levels **(D)**, leptin **(E)**, and non-esterified fatty acids (NEFA) **(F)**, in rats fed SC or a CAF diet and treated with Veh or ACT. Horizontal bars represent the mean ± s.e.m. (*n* = 9–11 per group). ^*^p < 0.05 by *t*-test. (*SC*, standard chow; *CAF*, cafeteria diet; *Veh*, vehicle; *ACT*, ACT-335827; *HDL*, high density lipoprotein; *ns*, not significant).

Plasma markers of MetS include reduced levels of HDLc and elevated TG levels. At the time of sacrifice following 4 weeks of vehicle or ACT-335827 treatment (and 18 weeks of diet exposure), plasma levels of HDLc (Figure 8A), total cholesterol (Figure 8B), or the proportion of HDLc relative to total cholesterol (Figure 8C) were not different between vehicle treated rats fed CAF diet or SC. However, plasma TG levels were significantly higher in vehicle treated CAF diet fed rats compared to SC fed rats [*t*_(18)_ = 2.70, *p* < 0.05; Figure 8D]. As expected from elevations in WAT and iBAT mass due to CAF diet feeding reported above, plasma levels of leptin were significantly elevated in these rats compared to SC fed rats [*t*_(18)_ = 2.72, *p* < 0.05; Figure 8E]. Finally, plasma levels of NEFA did not differ significantly between vehicle treated rats fed SC or CAF (Figure 8F).

ACT-335827 treatment of CAF diet fed rats did not significantly affect plasma levels of TG [*t*_(18)_ = 0.61, *p* = 0.55], cholesterol, NEFA, Plasma HDLc or leptin compared to vehicle treatment. However, the proportion of HDLc in the total plasma cholesterol was found significantly elevated [*t*_(18)_ = 3.84, *p* < 0.05; Figure 8C].

## Discussion

### CAF diet feeding as a model of MetS

In humans, over-consumption of palatable food rich in fat, sugar, and salt facilitates MetS development. Under a CAF diet comprised of a choice between a HF/S diet and SC and an additional snack 4 times weekly, rats developed major signs of human MetS, including abdominal adiposity, elevated TG and fasting blood glucose levels, glucose intolerance, and elevated blood pressure when compared to rats fed only SC. Conversely, plasma levels of NEFA, total cholesterol, HDLc, and the ratio between the latter two, were not altered. These findings are in agreement with those of other DIO studies in rodents reporting a similar induction of partial abnormalities associated with MetS in man [e.g., (Sampey et al., 2011)]. An impairment of hippocampus-dependent memory function induced by DIO, which might be a consequence of altered blood-brain barrier integrity (Davidson et al., 2012) and/or a reduction of growth factors and proteins responsible for synaptic plasticity (Woo et al., 2013), has been demonstrated both in human and rodents (Kanoski and Davidson, 2011). Still, hippocampus memory function, which was assessed in the present study by employing a CFC paradigm, was not altered in rats fed a CAF diet compared to rats fed SC. Using a one-trial conditioning protocol and a shock intensity similar to ours, Hwang et al. showed that male mice fed a normal diet responded with ~50% freezing during the 6 min test of conditioned fear, whereas this was reduced to ~20% in mice fed a HF diet for 1 year (Hwang et al., 2010). A CAF diet exposure of only 4 months as in our study may have been insufficient to cause learning impairments in this test.

### Effect of chronic act-335827 treatment in the MetS model

ACT-335827 treatment began in week 14 of CAF diet exposure, a time point when rats displayed elevated fasting blood glucose and plasma TG levels. Our results indicate that 4 weeks of ACT-335827 treatment did not significantly alter metabolic abnormalities and adiposity generated by CAF diet exposure alone, despite the animals showing an improved diet composition due to a slight but significant reduction in preference for the HF/S diet over SC. This reduction in HF/S diet intake upon OXR-1 blockade is consistent with other studies in rats suggesting a role for OXR-1 activation in positively regulating the motivation to consume palatable foods (Zheng et al., 2007; Choi et al., 2010). A decrease in reward perception under ACT-335827 treatment might also be expected to decrease intake of highly palatable snacks. However, consumption of the human snacks (presented for 1 h, 4 times weekly), was not affected by the drug. ACT-335827 administration may thus, be ineffective at reducing the binge-like intake of highly palatable foods under conditions, where either another type of palatable food (the HF/S diet) is freely available, or where no additional intermittent periods of stress or food-restriction occur. Using the OXR-1 antagonist GSK1059865, Piccoli et al. showed that OXR-1 signaling is only involved in the binge-like intake of highly palatable food if an additional specific stressor component (e.g., smelling but no access to the palatable food) is present (Piccoli et al., 2012).

The reduced overall food intake among all experimental groups during weeks 3 and 4 of vehicle or ACT-335827 treatment may be due to the performance of the CFC test to assess cognition (in week 3), and a blood pressure analysis and an overnight period of fasting before the oGTT (in week 4). These procedures were likely a source of stress on the rats, which may have affected their energy consumption and led to a slight decline of body weight gain in week 4. It is unlikely that those procedures confounded the overall conclusions of the current study with regard to OXR-1 blockade because all experimental groups (SC-Veh, CAF-Veh, and CAF-ACT) were simultaneously affected. Moreover, the overall pattern of the different diet consumption, which was displayed by the different groups already in week 2 of chronic treatment (until when no additional potentially stressful experimental procedures had been performed) was actually maintained in weeks 3 and 4 as well (see Figure 3A). Whether the additional exposure to environmental stressors during weeks 3 and 4 actually intensified the preference of the CAF-ACT treated rats for consuming more of the SC as compared to the CAF diet, cannot be excluded.

Despite affecting HF/S food preference vs. SC, ACT-335827 had no impact on total calorie intake because the decrease in HF/S food intake was entirely compensated for by an increase in SC intake. As a result, given that chronic ACT-335827 treatment also increased body weight gain, feed efficiency was enhanced. Although not directly tested, these results suggest that ACT-335827 administration may reduce energy expenditure. Funato et al. (2009) showed that energy expenditure is reduced in OXR-1 deficient mice under low fat, but not high fat, feeding conditions. Conversely, ob/ob female mice showed even increased energy expenditure under chronic treatment with SB-334867 administered at 30 mg/kg (Haynes et al., 2002). The reason for these discrepancies remains unclear, but biological compensation, phenotypically expressed in knockout animals, and sex or species differences are likely explanations that can be considered. Moreover, the OXR-1 preferential antagonist SB-334867 has recently been tested by two independent groups for selectivity (Gotter et al., 2012; Morairty et al., 2012): these studies suggest that at high doses as applied by Haynes et al. (2002), SB-334867 could additionally interact with a number of other brain targets.

Orexins also stimulate BAT thermogenesis, but the individual role played by the OXR-1 in modulating BAT function is unclear (Haynes et al., 2002; Sellayah et al., 2011; Tupone et al., 2011). BAT is important for plasma TG clearance, which improves insulin resistance due to DIO (Bartelt et al., 2011). In the current study the elevated weight gain and feed efficiency seen with ACT-335827 treatment under CAF diet feeding was not associated with an altered elevation in iBAT mass or a change in plasma TG levels compared to vehicle treatment. This might suggest that BAT was functioning normally in ACT-335827 treated rats challenged with a CAF diet. However, UCP-1 levels, which serves as a marker of BAT thermogenesis (Haynes et al., 2002; Sellayah et al., 2011), were not analyzed to confirm this conclusion. An alternative possibility for the reduced energy expenditure is that ACT-335827 had an inhibitory effect on non-exercise activity thermogenesis, which can be generated by orexin A (Novak et al., 2006; Novak and Levine, 2009).

Interestingly, the elevated body weight gain and increased feed efficiency seen over 4 weeks of ACT-335827 treatment were not associated with an increase in visceral fat mass compared to vehicle treatment. Thus, the weight gained by ACT-335827 -treated-rats must have occurred in fat deposits not analyzed (e.g., subcutaneous) and/or in non-fat tissues. Blood pressure of CAF diet fed rats also remained unchanged by treatment.

Rats exposed to a CAF diet for 13 weeks marginally consumed less water than rats fed SC in the 48 h before vehicle or ACT-33527 treatment onset; this decrease became significant over the subsequent 4 weeks of vehicle administration. ACT-335827 treatment, however, countered this decrease, even though acute administration of ACT-335827 to rats under normal feeding conditions does not affect water intake during their active phase (Steiner et al., 2013).

The mechanisms by which orexins modulate water balance or glucose metabolism through OXR-1 and OXR-2 binding have not been extensively investigated. Existing evidence suggests that orexins induce glucose production in the liver (Yi et al., 2009) and facilitate glucose uptake in skeletal muscle (Shiuchi et al., 2009). In addition, orexins A and B have been shown to differentially modulate glucagon release from pancreas (Goncz et al., 2008; Adeghate and Hameed, 2011). The unaltered glucose homeostasis observed with chronic ACT-335827 treatment in CAF diet fed rats with existing glucose imbalance contrasts with the improved fasting glucose levels seen in obese OXR-1 deficient mice (Funato et al., 2009) and in ob/ob female mice chronically treated with a high dose of the preferential OXR-1 antagonist SB-334867 before obesity onset (Haynes et al., 2002). Again, developmental compensation, species differences or non OXR-1 related effects may be responsible for these discrepancies.

A final observation of our study was that CAF diet fed rats chronically treated with ACT-335827 had elevated HDLc to total cholesterol ratios. HDLc is cardioprotective due to its antioxidant, anti-inflammatory, and scavenging properties (Mooradian et al., 2008). HDLc has a complex metabolism, and elevated levels must be interpreted in context with the overall lipid profile (Mooradian et al., 2008). To our knowledge there has been no reported involvement of the orexin system in HDLc processing; the mechanism by which ACT-335827 affects HDLc metabolism warrants further examination.

It is difficult to directly compare the results that we obtained using ACT-335827 with previous investigations using other OXR-1 antagonists, because this is the first study exploring chronic pharmacological OXR-1 blockade in a rat model of DIO associated with MetS. It also has to be mentioned that most of our knowledge concerning the effects of OXR-1 inhibition on food intake in rodents derives from using the OXR-1 antagonist SB-334867, which at high dose is likely to affect additional neuronal targets (Gotter et al., 2012; Morairty et al., 2012). Another newer generation OXR-1 selective compound, GSK1059865, for instance did not confirm previous findings using SB-334867 (Choi et al., 2010) in the sense that GSK did not affect high palatable food intake *per se* under no-stress and no food restriction conditions (Piccoli et al., 2012). Thus, it is desirable that future studies exploring the role of OXR-1 signaling in feeding further will employ not only SB-334867 but also other OXR-1 antagonists from different chemical classes.

Taken together, utilizing a free choice nutrient regime with additional human snacks as a model of DIO, this study was successful in inducing most aspects of human metabolic syndrome in Wistar rats. Chronic ACT-335827 treatment for 4 weeks reduced the preference for a high-fat/high-sweet diet compared to standard laboratory food pellets but had no effect on snack intake or absolute energy intake. ACT-335827 even slightly increased body weight gain. The main characteristics of human metabolic syndrome, including abdominal obesity, decreased glucose tolerance, enhanced blood pressure, and increased TGs remained unaffected by ACT-335827 treatment, at doses otherwise active in reducing environmental stress-induced outcomes (Steiner et al., 2013). It is concluded that continuous selective pharmacological blockade of OXR-1, in brain and periphery, under the applied experimental conditions has minimal impact on rat net energy balance resulting from food reward, nutrient metabolism, and physical homeostasis.

## Author contributions

Michel A. Steiner conceived, designed and supervised the study and wrote part of the manuscript. Carla Sciarretta and Anne Pasquali designed and planned the study, performed the experiments, and collected and analyzed the data. Carla Sciarretta also wrote a large part of the manuscript, and Anne Pasquali performed the statistical analyses. Francois Jenck facilitated and supported the study and critically reviewed the manuscript.

### Conflict of interest statement

All authors are employees of Actelion Pharmaceuticals Ltd.
