# Supplementary material for: The selective orexin receptor 1 antagonist ACT-335827 in a rat model of diet-induced obesity associated with metabolic syndrome
Source: Front Pharmacol. 2013 Dec 30;4:165. doi: 10.3389/fphar.2013.00165 (PMC3874552; doi:10.3389/fphar.2013.00165)
Supplement: Supplementary file 1 [file DataSheet1.PDF]

Supplementary Figure 1

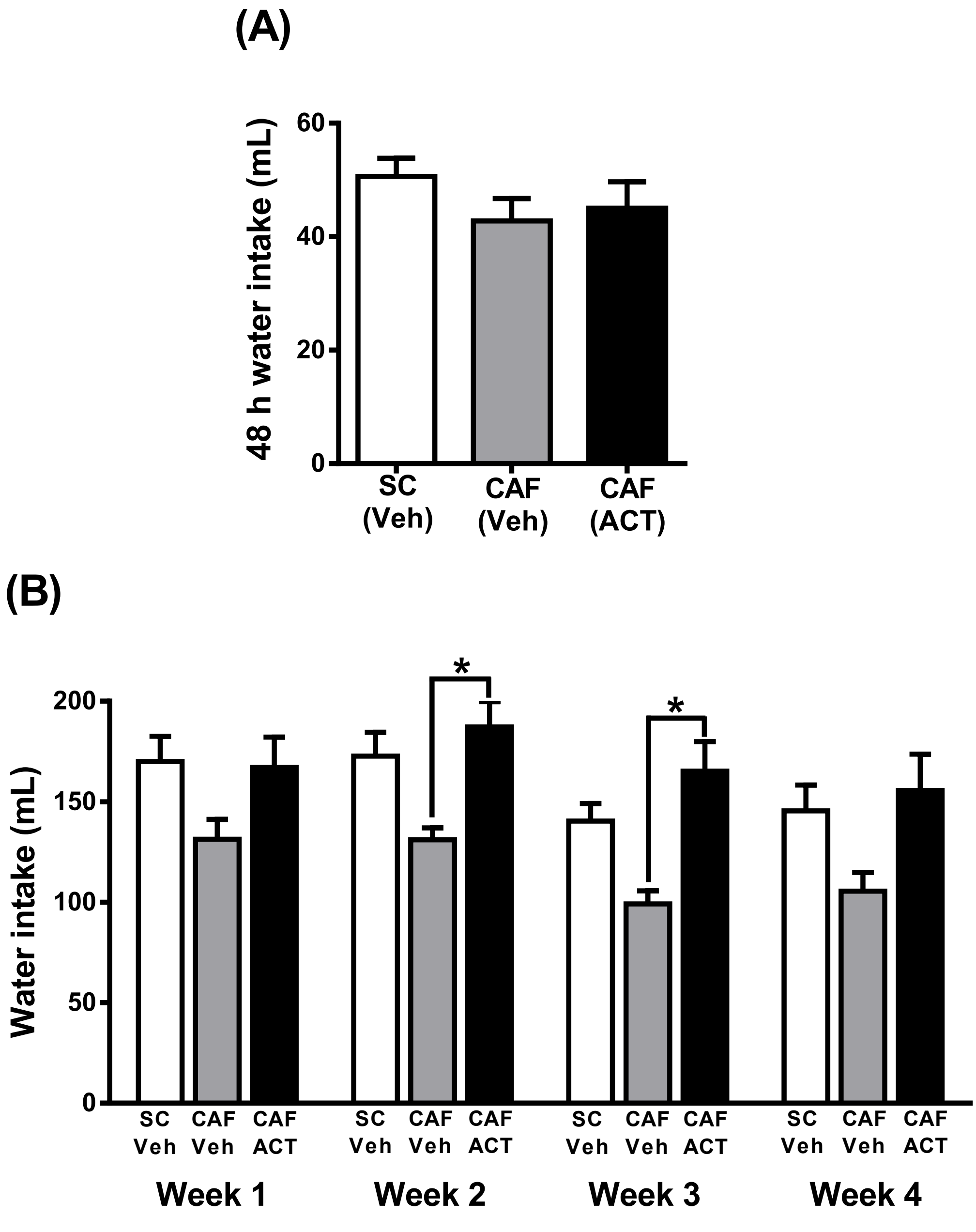

Supplementary Figure 1 Effect of diet and chronic ACT-335827 treatment on water consumption. A. Water intake of the experimental groups cumulated over 48 h before the start of vehicle or drug treatment (indicated in brackets). **B.** Cumulated weekly water intake of rats fed SC or CAF diet and treated with either Veh or ACT. *$p<0.05$ by post-hoc test following ANOVA; mean ± SEM; (n=9-11 per group). (*SC*, standard chow; *CAF*, cafeteria diet; *Veh*, vehicle; *ACT*, ACT-335827)
