# Supplementary material for: The selective orexin receptor 1 antagonist ACT-335827 in a rat model of diet-induced obesity associated with metabolic syndrome
Source: Front Pharmacol. 2013 Dec 30;4:165. doi: 10.3389/fphar.2013.00165 (PMC3874552; doi:10.3389/fphar.2013.00165)
Supplement: Supplementary file 2 [file DataSheet2.PDF]

Supplementary Table 1: Composition of presented snacks

Snack	Energy	Protein	Carbohydrate	Sugar	Lipids	Saturated fatty acids	Fiber	Sodium
Cheese/Bacon Crepes	5.64 kcal/g	7.8g (/100g)<br>31.2 kcal/100g<br>**5.53% kcal**	50g (/100g)<br>200 kcal/100g<br>**35.4% kcal**	15g<br>30% of Carbohydrate	37g (/100g)<br>333 kcal/100g<br>**59% kcal**	31g (/100g)<br>83.8% of Lipids	0.8g (/100g)	0.9g (/100g)
Salt Sticks	4.82 kcal/g	11g (/100g)<br>44 kcal/100g<br>**9.2% kcal**	60g (/100g)<br>240 kcal/100g<br>**49.8% kcal**	3.5g<br>5.8% of Carbohydrate	22g (/100g)<br>198 kcal/100g<br>**41.1% kcal**	14g (/100g)<br>63.6% of Lipids	3.5g (/100g)	1.1g (/100g)
Butter biscuits	4.71 kcal/g	5.3g (/100g)<br>21.2 kcal/100g<br>**4.5% kcal**	72.5g (/100g)<br>290 kcal/100g<br>**61.6% kcal**	36.9g<br>50.9% of Carbohydrate	17.7g (/100g)<br>159.3 kcal/100g<br>**33.8% kcal**	11.7g (/100g)<br>66.1% of Lipids	2.4g (/100g)	0.1g (/100g)
Chocolate biscuits	5.37 kcal/g	9g (/100g)<br>36 kcal/100g<br>**6.7% kcal**	55.4g (/100g)<br>221.6 kcal/100g<br>**41.3% kcal**	45.4g (/100g)<br>81.9% of Carbohydrate	31g (/100g)<br>279 kcal/100g<br>**52% kcal**	11.5g (/100g)<br>37.1% of Lipids	1.2g (/100g)	0.04g (/100g)
Honey balls cereal	3.85 kcal/g	6g (/100g)<br>24 kcal/100g<br>**6.2% kcal**	88g (/100g)<br>352 kcal/100g<br>**91.4% kcal**	23g (/100g)<br>26.1% of Carbohydrate	1g (/100g)<br>9 kcal/100g<br>**2.3% kcal**	<0.5g (/100g)<br><50% of Lipids	2g (/100g)	1.7g (/100g)
Choco Chip Cookies	4.97 kcal/g	7g (/100g)<br>28kcal/100g<br>**5.6%kcal**	61g (/100g)<br>244kcal/100g<br>**49%kcal**	34g (/100g)<br>55.7% of Carbohydrate	25g (/100g)<br>225kcal/100g<br>**45.3%kcal**	14g (/100g)<br>56% of Lipids	3.5g (/100g)	0.96g (/100g)
Big Butter biscuits	5.26 kcal/g	5g (/100g)<br>20kcal/100g<br>**3.8%kcal**	59g (/100g)<br>236kcal/100g<br>**44.9%kcal**	22g (/100g)<br>37.3% of Carbohydrate	30g (/100g)<br>270kcal/100g<br>**51.3%kcal**	15g (/100g)<br>50% of Lipids	2.5g (/100g)	0.69g (/100g)
